# The Relation of Serum Myeloperoxidase to Disease Progression and Mortality in Patients with Chronic Obstructive Pulmonary Disease (COPD)

**DOI:** 10.1371/journal.pone.0061315

**Published:** 2013-04-18

**Authors:** Hye Yun Park, S. F. Paul Man, Donald Tashkin, Robert A. Wise, John E. Connett, Nicholas A. Anthonisen, Don D. Sin

**Affiliations:** 1 UBC James Hogg Research Center and The Institute of Heart and Lung Institute, St. Paul’s Hospital, Vancouver, British Columbia, Canada; 2 Department of Medicine (Pulmonary Division), University of British Columbia, Vancouver, British Columbia, Canada; 3 Division of Pulmonary and Critical Care Medicine, Department of Medicine, Samsung Medical Center, Seoul, S. Korea; 4 Department of Medicine, David Geffen School of Medicine, University of California Los Angeles, Los Angeles, California, United States of America; 5 Division of Pulmonary and Critical Care Medicine, Johns Hopkins University, Baltimore, Maryland, United States of America; 6 Division of Biostatistics, School of Public Health, University of Minnesota, Minneapolis, Minnesota, United States of America; 7 Department of Medicine, University of Manitoba, Winnipeg, Manitoba, Canada; Clinica Universidad de Navarra, Spain

## Abstract

Myeloperoxidase is a strong oxidant stored in primary granules of neutrophils with potent antibacterial and proatherogenic properties. Myeloperoxidase has been implicated in the pathogenesis of chronic obstructive pulmonary disease (COPD). However, the relationship of myeloperoxidase to health outcomes in COPD is not well known. We measured serum myeloperoxidase levels from 4,677 subjects with mild to moderate airflow limitation in the Lung Health Study. Using a Cox proportional hazards model, we determined the relationship of serum myeloperoxidase concentration to the risk of all-cause and disease specific causes of mortality. We found that serum myeloperoxidase concentrations were significantly related to accelerated decline in forced expiratory volume in 1 second (FEV1) over 11 years of follow-up (p<0.0001), and this association persisted after adjustments for age, sex, race, baseline FEV1, and smoking status (p = 0.048). Serum myeloperoxidase concentrations were also associated with increased risk of cardiovascular mortality (p = 0.036). Individuals in the highest quintile of myeloperoxidase had a hazard ratio of cardiovascular mortality of 1.90 (95% confidence interval 1.00–3.58; p = 0.049) compared with those in the lowest quintile, which was particularly notable in patients who continued to smoke (adjusted p-value of 0.0396). However, serum myeloperoxidase concentration was not related to total mortality, respiratory mortality, or deaths from malignancies. In conclusion, increased serum myeloperoxidase levels are associated with rapid lung function decline and poor cardiovascular outcomes in COPD patients, which support the emerging role of myeloperoxidase in the pathogenesis of COPD progression and cardiovascular disease.

## Introduction

Chronic obstructive pulmonary disease (COPD) is a major global health problem, affecting over 10% of the population aged 40 years and older [1] and contributing to 3 million deaths annually [2]. A hallmark of COPD is airflow limitation, which is associated with persistent and progressive airway inflammation [3]. Interestingly, COPD is also associated with low-grade systemic inflammation, which in turn is related to accelerated decline in lung function and extra-pulmonary complications and co-morbidities such as cardiovascular disease and skeletal muscle dysfunction [4]–[6]. Numerous observational cohort studies have evaluated various inflammatory mediators including interleukin (IL)-6, IL-8, fibrinogen, Club cell secretory protein-16, tumor necrosis factor alpha (TNF-α) and white blood cell counts for their possible role in COPD progression and mortality [4], [6]–[9]. While there is growing consensus that neutrophils play a prominent role in the pathogenesis of COPD, the role of various enzymes expressed by neutrophils has not been well studied as possible circulating biomarker in COPD. One intriguing enzyme is myeloperoxidase (MPO). MPO is a lysosomal enzyme that neutrophils use to kill microbes by generating a potent oxidant, hypochlorous acid [10]. It is heavily expressed in primary (azurophilic) granules of neutrophils. A recent *in vivo* study has suggested a prominent role of MPO in the pathogenesis of COPD including progression of emphysema, pulmonary hypertension, and small airway remodeling [11]. In addition to its antimicrobial action, MPO also has potent proatherogenic properties and elevated serum levels of MPO have been associated with acute coronary syndromes [12], [13]. Cardiovascular diseases (CVD) are frequent and important co-morbidities in COPD [14]. However, the clinical relationship of MPO to progression and health outcomes in COPD is not well known. Using a large cohort of COPD patients [15], we assessed whether serum MPO is associated with an accelerated decline in lung function as measured by forced expiratory volume in 1 second (FEV1), and important COPD outcome such as total mortality and CVD mortality.

## Methods

### Subjects

We used data from the Lung Health Study (LHS) cohort. The details of this cohort have been published previously [16]. The original LHS study enrolled active smokers between the ages of 35 to 60 years who had mild to moderate airflow limitation on spirometry defined by FEV1 of <90% but ≥55% of predicted, in the presence of FEV1/forced vital capacity (FVC) ratio of <0.70 following bronchodilation. Individuals who had a history of cancer (except carcinoma in situ or basal-cell carcinoma of the skin), myocardial infarction (in the past two years), angina, heart failure, stroke (in the past two years), renal failure, insulin-requiring diabetes mellitus, cirrhosis or other serious liver diseases, pulmonary embolism, disorders of the central nervous system, narrow-angle glaucoma, or any other major diseases which could have compromised follow-up were excluded. After enrollment, the study participants were asked to visit the study center annually for 5 years. At these visits, salivary cotinine levels and exhaled carbon monoxide levels were measured to objectively verify smoking status of the participants. Participants were categorized as sustained quitters if they were biochemically validated nonsmokers at each annual visit. Participants who were smokers at each annual visit were defined as continuing smokers. Those whose smoking behavior varied were classified as intermittent quitters. At year 5 of follow-up, the participants were asked for a blood sample and approximately 89% of eligible participants provided informed consent for this procedure. The protocol of this study was approved by UBC/Providence Health Care Research Ethics Committee (No. H08-01864). All subjects provided written consent to participate in the study.

### Serum Sample Collection and Measurements

At the fifth annual visit, venipuncture was carried out on study participants. After collection, the blood samples were separated into their various components and transferred to the LHS data co-ordinating center on dry ice and were kept in −70°C freezers until use. The serum samples were thawed and MPO concentrations were measured using the SearchLight Proteome Array™ system (Pierce Biotechnology Inc, Rockford, IL). The details are published elsewhere [17].

### Lung Function Measurements

Spirometry was performed at the time of recruitment, annually for 5 years and then approximately 11 years following recruitment. We determined the rate of decline in lung function by taking the difference between baseline measurement at recruitment and the year 5 and between baseline measurement at recruitment and the year 11 and dividing it by the elapsed time between each two measurements (method 1). We also determined the rate of decline in lung function by taking into account all of the measured spirometric data for each of the study participants using a repeated measures modeling with PROC Mixed in SAS (method 2). As the results from this more complex model were similar to those of the simpler linear regression model, for parsimony, the primary analysis is based on method 1.

### Passive Follow-up

At year 5 visit, the participants were also asked to consent for additional follow-up (LHS 3). Participants were followed for additional 7 to 8 years during which their vital status data were captured biannually. An independent mortality and morbidity committee reviewed death certificates, autopsy reports, relevant hospital records, and summaries of interviews with attending physicians, or eyewitnesses and assigned the causes of death for all participants who died during the study. These data were supplemented by linkages with a National Death Index, which provided the date and cause of death for all U.S. study participants through the end of 2001. Vital status was successfully determined for 98.3% of the participants. Mortality end points were classified into: lung cancer, other cancers, respiratory diseases excluding lung cancer, cardiovascular disease (CVD), others, and unknown.

### Statistical Analysis

To evaluate the relationship of serum MPO to 1) total mortality; 2) disease-specific causes of mortality; and 3) FEV1 decline, we first divided the cohort into quintiles of MPO. The baseline characteristics across the quintiles were compared using analysis of variance (ANOVA) for continuous variables and a chi-square test for dichotomous variables. We used a Cox proportional hazards model to assess the relationship of MPO quintiles to the mortality endpoints using quintile 1 (lowest values) as the referent. Statistical adjustments were made for age, sex, body mass index (BMI), and race. The proportional hazards assumption was checked visually and it was met. To determine the robustness of the analysis, we repeated the analysis using serum MPO as a continuous variable rather than in quintiles. For continuous measurements (FEV1 decline), we used ANOVA. All analyses were conducted using SAS® version 9.2 (SAS Institute, Cary, NC). P values less than 0.05 were considered significant.

## Results

### Clinical Characteristics of the Cohort

**Figure 1:**
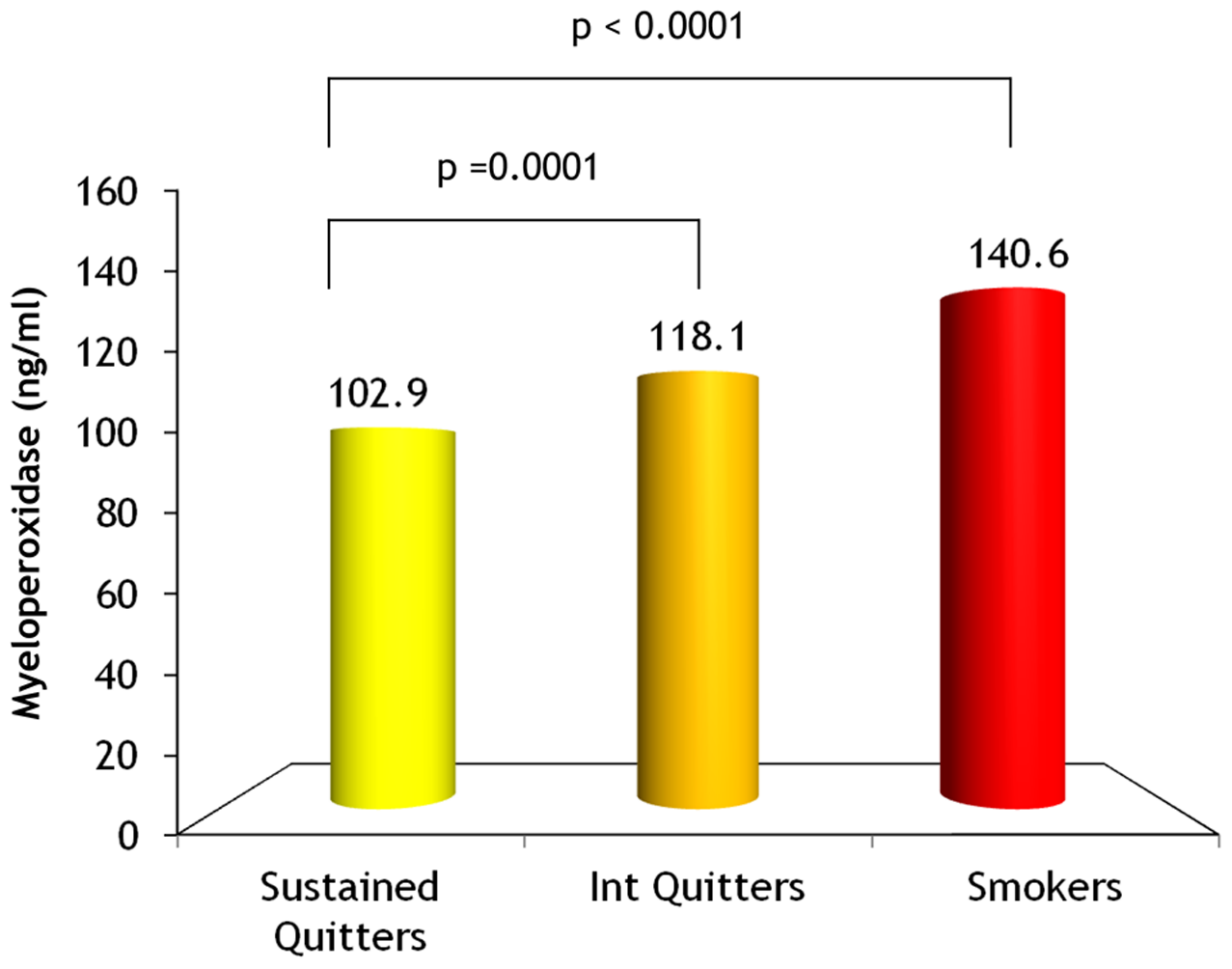
Myeloperoxidase level according to smoking status.

Serum MPO was measured in 4,677 LHS participants. Their demographic and clinical characteristics across quintiles of serum MPO concentrations are presented in Table 1. No significant differences were found between groups with regard to gender, BMI, race, and smoking duration. However, participants in the higher MPO quintiles were slightly younger and more likely to be continuing smokers compared to those with lower MPO levels. Even though baseline FEV1% predicted was similar across quintiles of serum MPO levels, FEV1% predicted at blood draw (year 5) and year 11 was inversely related to serum MPO levels. Serum CRP levels were positively associated with serum MPO levels. Continuing smokers had the highest serum MPO levels (140.6±126.7 ng/ml; p<.0001 versus sustained quitters), while sustained quitters had the lowest level (102.9±84.9 ng/ml) and intermittent quitters had moderate levels (118.1±95.3 ng/ml; p = .0001 versus sustained quitters) See figure 1.

**Table 1:** The Baseline Characteristics of the Lung Health Study Participants According to Quintiles of Myeloperoxidase Levels in Serum at Year 5.

	Quint1	Quint2	Quint3	Quint4	Quint5	P
Number	935	936	935	936	935	
Myeloperoxidase (ng/ml)	36.0±9.9	65.7±8.3	97.9±10.9	147.5±19.0	290.9±151.7	<.0001
Age (years)	54.2±6.7	53.3±6.9*	53.6±6.7	53.3±6.7*	52.7±6.9* †	0.0001
Men (%)	61.9%	62.0%	63.6%	62.8%	65.6%	0.4567
White (%)	97.0%	96.0%	95.0%	97.0%	97.0%	0.0660
BMI (kg/m^2^)	25.7±3.7	25.7±4.1	25.5±3.8	25.4±3.8	25.6±4.1	0.4357
Age at which smoking began (years)	17.7±4.3	17.6±3.9	17.5±3.8	17.4±3.6	17.3±3.5	0.0708
Smoking duration (pack-years)	39.8±18.8	39.6±18.8	40.1±18.4	40.2±18.6	40.8±18.5	0.5725
Sustained quitters (%)	22.6%	22.3%	19.7%	13.6%	10.1%	<.0001
Intermittent quitters (%)	32.2%	30.8%	25.9%	26.8%	25.7%	
Continuing smokers (%)	45.2%	46.9%	54.4%	59.6%	64.3%	
FEV1% Predicted at recruitment (year 0)	78.4±9.2	78.5±9.1	78.6±8.9	78.0±9.0	78.7±9.0	0.4372
FEV1% at blood draw (year 5)	76.1±12.1	75.6±12.2	75.1±11.7	73.8±12.3	74.6±12.0*	0.0005
FEV1% at year 11	70.7±14.5	70.1±15.3	68.8±14.6	68.3±15.4*	68.3±15.6*	0.0049
CRP (mg/L)	1.11 (1.66)‡	1.21 (2.01)‡	1.39 (2.39)‡	1.56 (2.78)‡	1.90 (3.50)‡	<.0001

Abbreviations: FEV1, forced expiratory volume in 1 second; BMI, body mass index. Study participants were divided into 5 identical groups based on myeloperoxidase levels. Data are presented as mean ± SD.

* p<.05 versus quintile 1.

† p<.05 versus quintile 2.

‡ p<.05 versus each other.

### Serum MPO and Health Outcomes of the Lung Health Study

**Figure 2:**
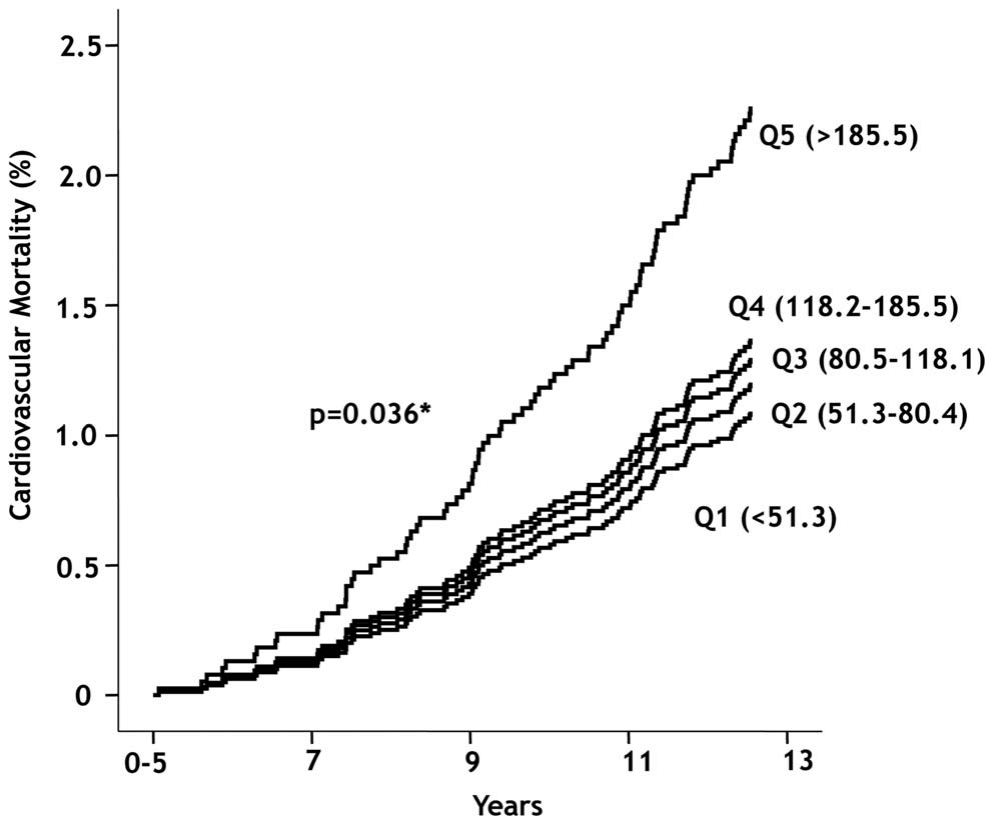
Cumulative cardiovascular mortality stratified to according to quintiles of myeloperoxidase levels. P value was derived from multivariable Cox proportional hazards model. The risk of cardiovascular mortality over the follow up period significantly increased along the increased quintiles of myeloperoxidase, after adjustments for age, sex, race and body mass index (p = 0.036).

As shown in Table 2, the rate of decline in FEV1 from year o to year 11 (ml/yr and % of predicted) significantly increased along the serum MPO quintile gradient (p<.0001 and p<.0001, respectively). During the 5-year period prior to measurement of serum MPO, 149 subjects died and more than half of the deaths were due to lung cancer (38.3%) and cardiovascular disease (24.8%) [15]. Of the 4,677 LHS participants, 445 subjects (9.5%) died during follow-up. 227 died from cancer (51.0%), 86 died from cardiovascular events (19.3%), and 42 died from respiratory causes (9.4%). Serum MPO concentration was not related to total mortality, respiratory mortality, or deaths from malignancies. However, the risk of cardiovascular mortality over the follow up period significantly increased along the MPO gradient, after adjustments for age, sex, race, and BMI (p = 0.036) (Figure 2). Most of the CVD mortality signal came from quintile 5. Compared with quintile 1, participants in quintile 5 had a hazard ratio of CVD mortality of 1.90 (95% confidence interval of 1.00 to 3.58; p = 0.049), adjusted for age, sex, race, and BMI. Quintile 2 had a HR of 0.91 (95% CI, 0.43 to 1.91; p = 0.798), quintile 3 had a HR of 1.14 (95% CI, 0.57 to 2.29; p = 0.705), and quintile 4 had a HR of 1.08 (95% CI, 0.53 to 2.21; p = 0.833). The “best” cutoff value to discriminate those who did and did not experience CVD mortality was determined by constructing a series of receiver operating characteristics (ROC) curves at various MPO values. The highest ROC statistics was observed at an MPO cutoff value of 210 ng/ml (ROC, 0.708; p<.0001).

**Table 2:** Health Outcomes of the Lung Health Study Participants According to Quintiles of Myeloperoxidase Levels in Serum at Year 5.

	Quint1	Quint2	Quint3	Quint4	Quint5	P for trend*
Total mortality	9.30%	8.55%	9.95%	9.83%	9.95%	0.1365
Cardiovascular mortality	1.60%	1.39%	1.82%	1.60%	2.78%	0.0359
Cancer mortality	5.67%	4.49%	5.24%	5.13%	3.74%	0.3696
Respiratory mortality	0.64%	0.96%	0.86%	1.07%	0.96%	0.3627
Decline in FEV1 from year 0 to year 11 (ml/yr)	44±33	44±34	49±33	52±33	52±35	<.0001
Decline in FEV1 from year 0 to year 11 (% of baseline FEV1)	16.8±12.4	16.9±13.0	18.2±12.3	19.3±13.4	19.2±13.2	<.0001

Abbreviations: FEV1, forced expiratory volume in 1 second.

* adjusted for age and sex and race and body mass index.

To determine whether the risk was modified by cigarette smoking, we performed stratified analysis according to subject’s validated smoking status at the time of blood draw. The most significant relationship was observed in the continuing smokers (adjusted p-value of 0.0396), see Table 3. The relationship between serum MPO and CVD mortality was not significant in the sustained or intermittent quitters.

**Table 3:** The Risk of Cardiovascular Mortality According to Quintiles of Myeloperoxidase Levels and Smoking Status.

	Serum Myeloperoxidase Levels					
Smoking Status	Quint1	Quint2	Quint3	Quint4	Quint5	P for trend*
Sustained quitters	1.42% (1)	0.96% (0.75)	1.09% (0.84)	0.79% (0.59)	1.06% (0.88)	0.7690
Intermittent quitters	1.33% (1)	1.39% (1.14)	0.83% (0.59)	0.80% (0.67)	1.67% (1.28)	0.9622
Continuing smokers	1.89% (1)	1.59% (0.91)	2.55% (1.35)	2.15% (1.26)	3.49% (2.09)	0.0396

Data presented as % of quintile column total (hazard ratio relative to quintile 1 adjusted for age, sex, race and body mass index).

* adjusted for age and sex and race and body mass index.

### Serum MPO and Lung Function

Serum MPO levels were significantly related to rate of decline in FEV1 over 11 years of follow-up (Table 2), after adjustments for age, sex, race, baseline FEV1 and smoking status, regardless of how FEV1 decline was calculated (Table 4). However, the overall effects of MPO on lung function declines were relatively small.

**Table 4:** The Rate of Decline in FEV1 According to Serum Myeloperoxidase LevelsOver 11 Years in LHS.

	Serum Myeloperoxidase Levels					
	Quint1	Quint2	Quint3	Quint4	Quint5	P for trend
Annual Decline in FEV1 from year 0 to year 11 (ml/yr, mean±SD)	44±33	44±34	49±33	52±33	52±35	<.0001
Annual Decline in FEV1 from year 0 to year 11 (% of baseline FEV1 mean±SD)	16.8±12.4	16.9±13.0	18.2±12.3	19.3±13.4	19.2±13.2	<.0001
Annual Decline in FEV1 from year 0 to year 11 (% of predicted per year, mean±SD)	0.74±1.03	0.76±1.07	0.89±1.03	0.93±1.09	0.94±1.11	<.0001
Adjusted Relative Rate of Decline in ml/yr (mean±SE)	1 (reference)	−0.37±1.62	2.01±1.61	3.67±1.63	1.62±1.64	0.0479*

A positive number denotes faster decline in FEV1.

* adjusted for age, sex, race, baseline FEV1, and smoking status at visit 5.

## Discussion

To our knowledge, this is the first investigation that has investigated the relationship of serum MPO concentrations to cardiovascular mortality and lung function decline over 11 years in a very large cohort of COPD patients with mild to moderate airflow limitation. We showed that serum MPO levels are related to accelerated decline in lung function and to increased risk of cardiovascular mortality particularly among those who continued to smoke.

MPO is an interesting molecule in COPD for several reasons. First, MPO is a very potent oxidant. In the presence of its substrates such as hydrogen peroxide and chloride, MPO catalyzes the formation of hypochlorous acid/hypochlorite (HOCl/OCl^-^), which is a powerful chlorinating oxidant with potent bactericidal and viricidal activities [10], [18]. These MPO-generated oxidants are capable of oxidizing a wide variety of compounds including products of cigarette smoke, and suborn tissue and vascular injury [10], [11]. Second, MPO is the principal enzyme stored in the azurophilic granules of neutrophils and is released upon their activation. Thus, serum and sputum MPO expression has been used as a surrogate marker of neutrophilic inflammation [19]–[23]. Third, the biological role of MPO in COPD has been recently elucidated *in vivo*. Using a guinea pig model of COPD, Churg and colleagues showed that inhibition of MPO with a small molecule can halt the progression of smoke-induced pathologic and physiological changes of COPD including emphysema, small airway remodeling and pulmonary arterial remodeling even when the drug was provided 3 months following smoking initiation [11].

Our data are consistent with a previous small study, which showed that sputum MPO expression in COPD patients predicted a rapid decline in FEV1 over 4 years [24]. However, this study evaluated fewer patients than 50 patients and some had bronchiectasis in addition to COPD. We extend the findings of this study to a much larger cohort of patients with prolonged follow-up. Our findings are also consistent with previous studies that have shown a positive relationship between serum MPO levels and poor cardiovascular health outcomes. For example, a previous large study has shown that serum MPO levels are associated with acute coronary syndromes among those who present to emergency departments with acute chest pain [13]. Other studies have shown that serum MPO levels can predict incident risk of cardiovascular disease and death in healthy middle-aged and elderly subjects [25], [26]. We extend these previous observations by demonstrating that serum MPO levels are related to incident cardiovascular mortality in COPD patients who continue to smoke. While causality cannot be established on our study, these data nonetheless suggest that neutrophilic inflammation may be very important in the pathogenesis of COPD-related cardiovascular outcomes. It should be noted that the mechanisms underlying the relationship between MPO and cardiovascular outcomes may be different than that between MPO and FEV1 decline. Additional studies will be needed to sort out the mechanistic pathways relevant to these relationships.

There are several limitations to this study. First, the LHS cohort recruited only patients with mild or moderate disease. It is uncertain that these results can be generalized to COPD patients with more advanced disease. Second, we did not have multiple measurements of lung function after blood sample analysis, and potential factors such as the frequency of acute exacerbation contributing to rapid progression in COPD patients could not be explored in this study. Third, since only one venipuncture was carried out on the fifth annual visit during the study period, we could not evaluate intra-and inter-individual variation of serum MPO over time in patients. Moreover, we could not ascertain MPO levels in those who died or were lost to follow-up over the first five years of the study. As we did not have serial measurements of MPO over time, it is possible that some in quintile 5 became quintile 1 members and vice versa, resulting in misclassification of exposure status over time and thus diluting the relationship of serum MPO to FEV1 progression and mortality. Thus, our estimates may be conservative. Moreover, Agusti et al recently showed that 70% of patients with COPD who failed to demonstrate systemic inflammation at baseline remained “free” of systemic inflammation at 1 year follow-up, while 80% of patients with systemic inflammation (defined as having two or more elevated blood biomarkers) at baseline continued to demonstrate an inflammatory phenotype at follow-up [27]. Most importantly, patients with systemic inflammation had poor clinical outcomes including worse health status and increased risk of exacerbations. We extend the findings of this study showing that serum myeloperoxidase is a promising biomarker for tracking rate of decline in lung function over time and in risk-stratifying patients for cardiovascular mortality. Fourth, lung function measurements were made before and after venipuncture and as such the directionality of the relationship between serum MPO and decline of FEV1 is uncertain. It is possible that serum MPO “caused” a rapid decline but reverse causation was also possible. Furthermore, although serum MPO levels were significantly associated with accelerated decline in FEV1, the overall effect was very small and of questionable clinical significance. Fifth, although we assessed disease progression using FEV1 decline data, it should be noted that this end point is highly variable among patients with COPD [4], [28], [29] with a low signal to noise ratio. As such, the use of other measurements (such as diffusing capacity or computed tomography based lung densitomety) would have strengthened the findings. Finally, while promising, our data cannot be considered definitive as LHS was not originally designed to identify novel blood biomarkers for COPD and the analysis (by definition) had to be retrospective in nature. A prospective study would be needed to validate these promising findings.

In summary, we found that serum MPO levels were significantly related to rapid lung function decline and poor cardiovascular outcomes in COPD patients, which is in line with the hypothesis that MPO plays an active role in the pathogenesis of COPD progression and cardiovascular disease.
